# Quality of high-energy X-ray radiotherapy beams: Issues of adequacy of routine experimental verification

**DOI:** 10.4103/0971-6203.39416

**Published:** 2008

**Authors:** S. D. Sharma

**Affiliations:** Radiological Physics & Advisory Division, Bhabha Atomic Research Centre, CT & CRS, Anushaktinagar, Mumbai - 400 094, India E-mail: sdsbarc@gmail.com

Medical electron linear accelerator is an important equipment used in radiotherapy departments worldwide. Measurement of beam quality of high-energy X-rays generated by a medical electron linear accelerator (LINAC) is required for three different purposes, namely, (i) to verify the stated specification of the vendor, (ii) to determine the appropriate beam-quality–correction factor for the ionization chamber and (iii) to determine the shielding thickness of the primary and secondary barriers of the accelerator housing. Recently, a few instances of significant mismatch between quoted nominal X-ray-beam energy (>6 MV) and measured X-ray–beam energy have been noticed by radiotherapy centers during acceptance and commissioning of newly installed medical LINACs; therein, it is important to note that one of the beam-quality indicators (TPR_20,10_) indicated a particular value of nominal X-ray energy while the measured dosimetry data (percentage depth dose) indicated a significantly different nominal energy. Such observations create confusion during the clinical use of the accelerator, and the user becomes unsure about whether to use the dosimetry data measured locally or to use the standard data set available in the literature corresponding to the measured quality of the X-ray beam. The significant variation in quoted and measured values of X-ray–beam energy also raises doubt over the adequacy of shielding thickness of the accelerator housing.

All the major manufacturers of medical electron linear accelerators (e.g., Elekta, Siemens, Varian) declare IEC 60977[1] compliance of their accelerators. Accordingly, the energy of the photon beam is specified in terms of nominal accelerator potential/ beam energy (MV or MeV), as well as in terms of a parameter which indicates penetrative quality of the beam. Commonly quoted penetrative quality indicator of the X-ray beam by the manufacturer is percentage depth dose at 10-cm depth (D_10_) for 10 × 10 cm^2^ field size at the phantom surface and 100-cm source surface distance (SSD) [e.g., Varian Medical Systems, USA, quotes D_10_ of their 10-MV X-ray beam as 74.0 ± 1.0%]. In addition, the manufacturer also quotes the depth of dose maximum (d_m_) as an alternative quality indicator of the beam. These two X-ray penetrative quality indicators suffer from the effect of electron contamination and may change their magnitude due to variation in components and accessories in the head of the accelerator.[1–4] AAPM has also adopted D_10_ as a quality-specifying parameter for X-rays under the recommendation that measurement should be done using 0.1-cm lead filter to limit the contribution of contamination electrons.[4] Other recommended X-ray beam quality indicators are depth of 80% dose (d_80_) for 10 × 10 cm^2^ field at an SSD of 100 cm along the central axis of the beam[5] and the ratio of tissue phantom ratio (TPR) at the depths of 20 and 10 cm (TPR_20,10_).[2,3,6] The method of d_80_ for energy specification also has a practical shortcoming related to electron contamination. The parameter TPR_20,10_ is a measure of the effective attenuation coefficient describing approximately the exponential decrease of X-ray depth–dose curve beyond d_m_ and is independent of the electron contamination in the incident beam.[2,3]

Table 1 lists the values of D_10_, d_80_ and TPR_20,10_ at different values of nominal energy (MV) of X-ray beams generated by medical LINACs. It can be observed from this table that 1.0% change in D_10_ value (>6 MV) results in about 1.0 MV corresponding change in nominal beam energy. This means the nominal X-ray energy specified by the vendor has a tolerance of about 1.0 MV. This in turn indicates that the exact matching of the X-ray–beam energy quoted by the vendor with the X-ray–beam energy measured by the user would definitely be a random coincidence and can be an issue of dispute. D_10_ and d_80_ do not show saturation with increasing nominal beam energy and hence can be regarded as sensitive beam-quality indicators. TPR_20,10_ shows saturation at higher beam energy (>12 MV), which is a major drawback of this parameter. As far as the use of TPR_20,10_ is concerned for determining MV of an X-ray beam, it would be an inferior choice. But for determining beam-quality conversion factor for ionization chambers used for dose rate calibrations of output of X-rays from medical LINACs, this parameter is found to be relatively more suitable.[2] It is also observed from the data in Table 1 that among the three recommended X-ray–beam quality indicators, d_80_ has comparatively higher sensitivity.

**Table 1:** Nominal energy (MV) and beam-quality indicator for high-energy radiotherapy X-rays[5,6]

*Nominal energy (MV)*	*Beam quality indicator*		
	
	*D_10_ (%)*	*d_80_ (cm)*	*TPR_20,10_*
6	67.50	6.70	0.676
8	71.00	7.50	0.703
10	73.00	8.00	0.724
12	75.00	8.50	0.740
15	77.00	9.10	0.755
18	79.00	9.70	0.771
21	81.00	10.30	0.777
25	83.00	10.90	0.781

Table 2 lists the values of tenth value thickness [both first TVL (TVL1) and equilibrium TVL (TVLe)] for ordinary concrete from NCRP Report 151.[7] These two TVLs are used to determine the absolute thickness of a radiation barrier [wall thickness of a barrier = TVL1 + (n-1) TVLe]. To determine the shielding thickness of primary and secondary barriers of the accelerator housing, it is important to determine the beam energy in terms of MV. However, determining the accelerating potential directly is not practical at the hospitals. Quality of the X-ray beam is measured in terms of D_10_ and d_80_ or TPR_20,10_, and correlation curves generated using data from Table 1 are used to calculate corresponding MV values. However, accuracy of MV value so determined depends on the accuracy and sensitivity of beam-quality indicator selected. Change in values of TVL with changing nominal beam energy, particularly for high-energy X-rays (>15 MV), is relatively small and hence an error of 1.0 MV or so in determining the nominal beam energy would not affect the shielding thickness drastically.

**Table 2:** Tenth value thickness for ordinary concrete at different values of X-ray–beam energy (The data quoted in this table are taken from NCRP Report 151)[7]

*Nominal energy, MV*	*Tenth value thickness (TVL) for ordinary Concrete (cm)*	
	
	*First TVL (TVL1)*	*Equilibrium TVL (TVLe)*
4	35	30
6	37	33
10	41	37
15	44	41
18	45	43
20	46	44

Dosimetry and quality assurance protocols recently in use recommend a number of parameters for qualifying beam quality for heterogeneous X-rays generated by medical LINACs. The choice of parameters for qualifying beam quality is governed by the objective of the given task. The parameter d_80_ measured under the electron-contamination–free condition as recommended by AAPM[4] for D_10_ will be a better choice for verifying manufacturer-specified nominal X-ray–beam energy and determining shielding thickness, as it shows an increasing nature with increasing nominal beam energy. However, for determining the beam-quality conversion factor for beam dosimetry purposes, protocol-specific beam-quality indicator should be measured and used. Dosimetry data generated following a standard technique at the hospital site should be used for patient dosimetry, irrespective of the difference in stated and measured energy of the X-ray beam.
